# Amino acid-based formula and *Clostridioides difficile*-associated disease in infants: concerns regarding causal interpretation

**DOI:** 10.1080/19490976.2026.2761714

**Published:** 2026-11-12

**Authors:** Xun Jiang, Ying Wang, Youyou Luo, Cuiyuan Jin, Zhaoyang Zeng, Yan Hu, Huan Chen, Sitang Gong, Jie Wu, Min Yang, the Subspecialty Group of Gastroenterology, the Society of Pediatrics, Chinese Medical Doctor Association

**Affiliations:** a Department of Pediatrics, Tangdu Hospital, Air Force Medical University, Xi’an, China; b Division of Pediatric Gastroenterology and Nutrition, Xinhua Hospital Affiliated to Shanghai Jiao Tong University School of Medicine, Shanghai, China; c Department of Gastroenterology, Children’s Hospital, Zhejiang University School of Medicine, Hangzhou, China; d Children’s Hospital, Zhejiang University School of Medicine, National Clinical Research Center for Children and Adolescents’ Health and Diseases, Hangzhou, China; e Key Laboratory of Artificial Organs and Computational Medicine in Zhejiang Province, Institute of Translational Medicine, Zhejiang Shuren University, Hangzhou, Zhejiang, China; f Department of Pediatrics, Guangdong Provincial People’s Hospital, Guangdong Academy of Medical Sciences, Southern Medical University, Guangzhou, China; g Department of Gastroenterology, Guangzhou Women and Children’s Medical Center, Guangzhou Medical University, Guangzhou, China; h Department of Gastroenterology, Beijing Children’s Hospital of Capital Medical University, Beijing, China

**Keywords:** *Clostridioides difficile*, amino acid-based formula, infant gut microbiota, fecal microbiota transplantation, causal inference

## Abstract

Wang et al. recently reported an association between amino acid-based formula (AAF) feeding and *Clostridioides difficile*-associated disease (CDAD) in children younger than two years, integrating clinical observations, microbiome profiling, and fecal microbiota transplantation experiments. The authors appropriately framed their findings as associative; our aim is not to allege over-interpretation, but to delineate the evidentiary gap between their reported association and a causal claim. Several methodological and interpretative issues limit such inference, including confounding by the clinical indication for AAF, the difficulty of distinguishing *C. difficile* colonization from clinically attributable disease in young children, developmental differences in age and complementary feeding, and the selected nature of the fecal microbiota transplantation (FMT) donor population. For example, the multivariable model included only 27 outcome events against approximately six predictors (events-per-variable ≈ 4.5, far below the accepted 10–20 threshold), rendering the adjusted odds ratios unstable. The cross-sectional microbiome data further cannot establish whether microbial alterations preceded disease or resulted from underlying clinical, dietary, or inflammatory states. We therefore suggest interpreting the findings as an association between AAF feeding and CDAD status, rather than evidence that AAF itself increases CDAD risk. Our skepticism concerns the causal link to CDAD specifically; we do not dispute that reduced *Bifidobacterium* with formula feeding is a well-established finding. This distinction has important clinical implications, as AAF remains an essential nutritional intervention for children with appropriate indications, and in young children microbiological detection and microbiome alterations must be interpreted alongside age, clinical symptoms, underlying disease, dietary history, and other relevant exposures. An overly causal interpretation could create unnecessary concern among families and complicate clinical decision-making. To make these considerations operational, we specify the elements of a decisive prospective study, including a minimum event count of ~60 adjudicated CDAD cases, a blinded tiered diagnostic algorithm, and detailed AAF exposure ascertainment.

## Introduction

Wang and colleagues recently published a study in Gut Microbes (2026;18:2719259) reporting a significant association between amino acid-based formula (AAF) feeding and increased risk of *Clostridioides difficile*-associated disease (CDAD) in children under two years of age.^1^ The study addresses an important and understudied clinical question and combines clinical observations, microbiome profiling, and fecal microbiota transplantation (FMT) experiments. The authors identified a robust association between AAF exposure and CDAD incidence; moreover, they demonstrated markedly reduced *Bifidobacterium* abundance in AAF-fed infants and increased susceptibility to *C. difficile* infection (CDI) in PEG-conditioned mice colonized with microbiota derived from AAF-fed infants.

At the outset, we note that Wang et al. framed their findings as associative, acknowledged that confounding by indication could not be excluded, and appropriately called for larger prospective studies. We also do not dispute their microbiome observations: reduced *Bifidobacterium* with formula feeding relative to breastfeeding is among the most replicated findings in infant gut microbiology. Our aim in this commentary is therefore not to allege over-interpretation by the original authors, but to delineate the evidentiary gap between their reported association and a causal claim, and to specify what additional evidence would close it. This distinction matters because several features of the study, while compatible with the authors' cautious framing, could be read by clinicians and families as establishing causation. A concrete example illustrates the gap: the multivariable analysis rests on 27 outcome events distributed across approximately six dietary predictors, an events-per-variable ratio of ~4.5 that is far below accepted thresholds, so the adjusted effect estimates carry considerable uncertainty.

Nevertheless, several methodological and interpretative considerations warrant attention when translating these findings into causal and clinical conclusions. In particular, confounding by the clinical indication for AAF, the difficulty of distinguishing *C. difficile* colonization from clinically attributable disease in young children,^2^ ^,^ ^3^ the developmental context of infant feeding,^4^ ^,^ ^5^ and the selected nature of the experimental FMT model may limit causal inference. We discuss these issues below and consider their implications for interpreting AAF-associated CDAD risk.

## Discussion

### Confounding by indication and the clinical context of AAF use

AAF is not a randomly assigned exposure. According to ESPGHAN and Chinese Society of Pediatrics guidelines, AAF is indicated for dietary replacement during the diagnostic period of suspected cow's milk protein allergy (CMPA), severe CMPA, multiple food allergies, or related clinical indications.^6^ ^,^ ^7^ These underlying conditions may themselves influence intestinal inflammation, mucosal function, dietary patterns, and gut microbial composition. Previous studies have also reported alterations in the gut microbiota, including reduced *Bifidobacterium*, in infants with CMPA.^8–10^

The authors acknowledge that confounding by indication cannot be entirely ruled out. However, adjustment for a binary history of food allergy may not fully capture the clinical heterogeneity underlying AAF prescription. Disease severity, gastrointestinal symptoms preceding AAF initiation, duration of AAF exposure, elimination diets, prior formula intolerance, and the timing of AAF initiation may all influence the observed microbial phenotype.^11^ Moreover, because AAF is available in China without prescription and is used by some parents for dyspeptic symptoms outside strict CMPA indications (a caveat Wang et al. themselves raised), the exposed population mixes true CMPA with a broader symptomatic population, making a single binary covariate even less adequate.

That potential confounders are difficult to eliminate by simple adjustment for a binary variable is a well-established methodological principle. Confounding by indication is a recognized source of spurious association in observational research, and single-covariate adjustment does not recover causal effects when the confounder is multi-dimensional (e.g., disease severity, healthcare utilization).^11^ Better-suited approaches include indication-stratified analyses, propensity-score weighting or matching constructed from the full pre-AAF clinical phenotype,^12^ ^,^ ^13^ and, most powerfully, within-child designs in which each patient serves as their own control. An instrumental-variable approach, for example exploiting regional variation in prescribing practice, offers a further option, although its identifying assumptions would be difficult to verify in a study of this size. We also recognize that the original study's modest sample size precludes most of these methods, which is itself a reason the association should not yet be read causally.

More complex causal pathways also deserve consideration: infants and toddlers with CMPA or other allergic conditions may receive more medical interventions, including antibiotic use or hospitalization, due to repeated clinic visits or nutritional problems, and these factors are known risk factors for CDAD, yet they could not be adequately assessed because of the limited sample size. Allergy-related changes in intestinal barrier function and immune regulation may independently affect susceptibility to *C. difficile* toxins. These potential confounders are difficult to eliminate by simple adjustment.

Wang et al. reported that approximately 29% of asymptomatic *C. difficile* carriers were also receiving AAF.^1^ This observation does not refute the possibility that AAF contributes to CDAD risk under certain conditions; it indicates that AAF exposure is not sufficient on its own and that additional clinical or ecological co-factors, such as disease activity, antibiotic use, or developmental stage,^14^ are likely necessary.

Thus, the central unresolved question is not simply whether AAF is associated with CDAD, but whether AAF itself is a causal determinant of disease susceptibility or instead identifies a clinically distinct population with other factors that influence both microbiota composition and CDAD risk.^15^

### CDAD, colonization, and the estimation of disease risk in young children

The clinical analysis compared 27 CDAD cases with 56 asymptomatic *C. difficile* carriers (a case-carrier design). Because controls were drawn from *C. difficile*-positive children rather than from the source population of all AAF-exposed infants, this design estimates the odds of symptomatic (rather than asymptomatic) status among tested carriers; it does not estimate the incidence of CDAD in AAF-exposed children, nor the population attributable fraction of AAF. The association it yields is therefore a statement about which colonized children become symptomatic, not about how often AAF-fed infants develop CDAD.^16^

This distinction is particularly important in young children because *C. difficile* colonization is common during infancy.^2^ ^,^ ^3^ ^,^ ^17^ The 2018 IDSA/SHEA guideline recommends against routine testing for *C. difficile* in infants ≤ 12 months because of the high prevalence of asymptomatic carriage, reported to reach 30%–70% in this age group.^18^ Testing may nevertheless be appropriate in selected infants with clinically significant diarrhea or severe manifestations after alternative causes have been considered.^18^

The present study defined asymptomatic carriers as children without diarrhea who had positive *C. difficile* cultures. In contrast, CDAD cases were children presenting with diarrhea, mucoid stool, and/or hematochezia, along with microbiological evidence of *C. difficile*. We do not contend that the reported cases were misclassified. Rather, given the high background prevalence of *C. difficile* colonization in this population, clinically attributing gastrointestinal symptoms to *C. difficile* remains challenging, particularly among AAF-fed infants whose underlying indications for AAF may themselves involve gastrointestinal manifestations. Designs that would better address incidence include a denominator-defined prospective cohort of AAF-exposed infants with feeding as a time-varying exposure, or a nested case-control study using incidence-density sampling of controls from the full source population;^19^ controls drawn only from *C. difficile* carriers would still estimate risk within the colonized population and could not yield population-level incidence. We return to this in the design section below.

The absence of a significant difference in Chao richness between CDAD cases and asymptomatic carriers (*P* = 0.971) does not, in itself, invalidate the diagnosis. However, it prompts reflection on whether the observed CDAD cases were characterized by the degree of microbiota disruption expected under a microbiota-depletion model of pathogenesis. Consequently, positive *C. difficile* detection should be interpreted together with age, symptom severity, underlying gastrointestinal disease, and exclusion of alternative causes.^20^ More detailed reporting of the clinical adjudication process and microbiological criteria would further strengthen confidence in the classification of CDAD in this age group.

### Developmental stage and complementary feeding as potential confounders

The CDAD group was significantly older than the asymptomatic carrier group (median 15 versus 11 months). During the first two years of life, age is closely linked to intestinal microbiota maturation, *C. difficile* colonization dynamics, introduction of complementary foods, and increasing dietary diversity.^4^ ^,^ ^5^ Consumption of dairy products, eggs, red meat, and fruit was negatively associated with CDAD, whereas AAF feeding was positively associated; these dietary variables are themselves developmentally correlated with age.^21^ ^,^ ^22^

Although the association between AAF feeding and CDAD remained significant after adjustment for age (OR 9.346, 95% CI 2.399–45.84, *P* = 0.003), the precision of this estimate must be interpreted in light of the model's dimensions. With only 27 outcome events and approximately six predictors in the multivariable model, the events-per-variable ratio is ~4.5, far below the rule-of-thumb threshold of 10–20, raising concern about instability, overfitting, and unreliable confidence intervals.^23^ This limitation applies specifically to the main multivariable model containing six dietary predictors. The age-adjusted estimate (OR 9.346) should be interpreted according to the number of covariates included in that model: if only age and AAF were entered, the events-per-variable ratio would be acceptable; however, the analysis remains underpowered for the full set of dietary predictors, and the width of the reported confidence interval (2.399–45.84) is consistent with considerable imprecision.

Feeding status was also defined according to the predominant nutritional source during the four weeks preceding stool collection. This relatively short exposure window may not adequately capture cumulative dietary history or the duration of AAF exposure, particularly when the outcome reflects longer-term processes. Prospective studies with longitudinal assessment of feeding history, age, complementary food introduction, duration of AAF exposure, and microbiome dynamics would therefore be valuable for clarifying the temporal relationship between diet, microbial composition, and CDAD.^24^

### What would a decisive prospective study look like?

To convert these criticisms into practical guidance, we outline the design elements that a decisive study would require (Figure 1). As a concrete anchor, these elements draw on an eight-center prospective study our group is currently launching (320 infants), which combines a cross-sectional comparison of stable AAF users with non-allergic formula-fed controls and an embedded within-child pre/post AAF-switch cohort with paired sampling at baseline and 4 weeks; five of the eight centers are led by the chair or vice-chairs of the Pediatric Gastroenterology Group of the Pediatric Society of the Chinese Medical Doctor Association, which facilitates standardized, blinded CDAD adjudication across regions. (1) Events and sample size: to model ~6 dietary and clinical predictors with an events-per-variable ratio ≥ 10, at least ~60 adjudicated CDAD events are required for the main model;^25^ if mediation,^26^ subgroup, or multiple exposure-window analyses are planned, 120–200 events would be prudent. A nested case-control design should sample controls by incidence-density sampling from the entire source population of AAF-exposed infants (not only from *C. difficile* carriers) and would require at least 60 cases plus matched controls, the precise total depending on the assumed CDAD incidence among colonized infants, the matching ratio, and anticipated missingness. Estimating a population attributable fraction would additionally require the population prevalence of AAF exposure. (2) An age-appropriate diagnostic algorithm: blinded expert adjudication using a tiered classification (*C. difficile*-negative, non-toxigenic carriage, toxigenic asymptomatic carriage, toxin-positive asymptomatic, possible CDAD, and confirmed CDAD requiring symptoms, toxigenic organism, toxin evidence, and exclusion of alternatives), operationalizes IDSA/SHEA guidance for this age group and separates colonization from disease far more rigorously than any single laboratory cut-off. (3) Exposure ascertainment: AAF indication, brand (including HMO or probiotic content), fraction of total milk intake, and cumulative duration from initiation should be recorded, replacing the 4-week predominant-feeding window. (4) Confounding control: a within-child pre/post AAF-switch cohort controls all time-invariant confounders by construction and directly tests whether AAF initiation changes *Bifidobacterium*, toxigenic *C. difficile* carriage, and fecal inflammatory markers within the same child. Within-child pre/post-switch designs of this kind, executed across multiple centers with standardized blinded adjudication, cannot by themselves prove absence of harm, but they can establish time-ordering and effect size with far greater credibility than a case-carrier comparison. Within this scheme, toxigenic asymptomatic carriage denotes detection of a toxigenic strain without symptoms, whereas toxin-positive asymptomatic denotes detectable free toxin without symptoms: two biologically distinct states that should not be conflated. Consistent with IDSA/SHEA, children ≤ 12 months would not be tested routinely, with testing reserved for severe or high-risk presentations.

**Figure 1.**
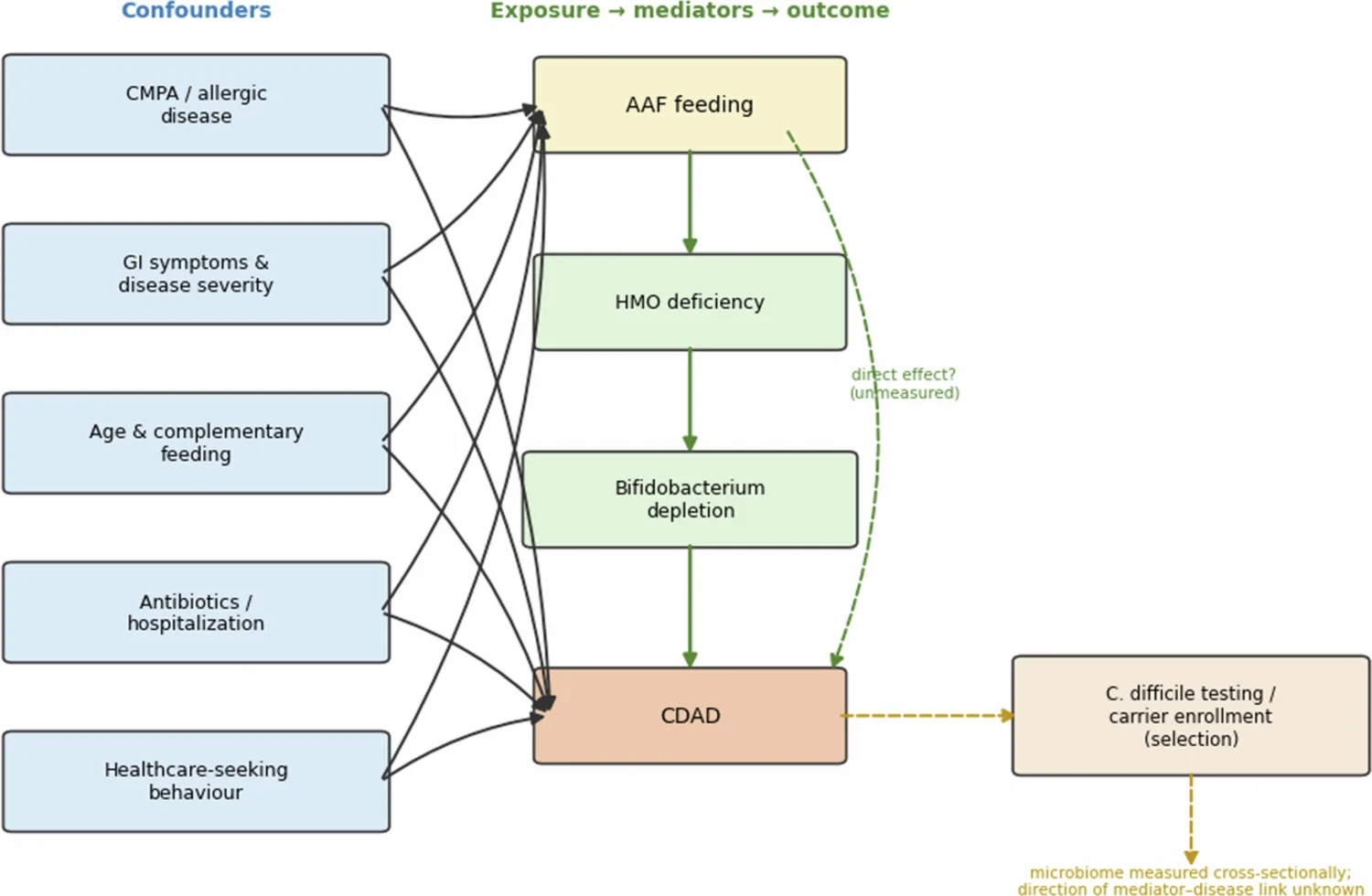
Directed acyclic graph summarizing the causal-inference framework of this commentary. Confounders (blue) influence both AAF prescription and CDAD risk; the proposed mediation chain (green) runs from AAF feeding through HMO deficiency and *Bifidobacterium* depletion to CDAD; conditioning on *C. difficile* positivity at enrollment (orange selection node) precludes estimation of population-level incidence, and cross-sectional microbiome sampling cannot establish the direction of the mediator–disease relationships (dashed arrows).

### Interpretation and generalizability of the FMT experiments

The FMT experiments provide an important functional component of the study. PEG-conditioned C57BL/6 mice were colonized with pooled bacterial consortia from healthy, *C. difficile*-negative AAF- or NF-fed infants under two years of age, then challenged with *C. difficile*. The AAF-associated consortium was associated with greater persistence of *C. difficile* and increased inflammatory infiltration, supporting the biological activity of the selected AAF-associated microbiota.

Several features of the donor selection limit what this experiment can establish about human causality. Donors were healthy, had not received complementary foods, and their samples were pooled to generate the AAF-BC and NF-BC communities. Pooling reduces experimental heterogeneity but obscures the substantial inter-individual variation among AAF-fed infants, prevents donor-level replication, and could allow one or two donors to drive the phenotype. Excluding complementary-fed donors is also consequential because complementary-food consumption was itself associated with lower CDAD occurrence in the clinical analysis; the experiment thus excludes a variable that may be biologically important rather than merely a confounder. We share the concern that the small number of animals per group limits statistical power.

The most fundamental limitation is the human-relevance gap. It must be demonstrated by careful analysis of its specific features rather than merely asserted by citation. PEG-mediated depletion of an adult mouse microbiome creates an ecological state very different from the infant intestine: bile-acid composition, epithelial toxin receptors, immune maturation, and the baseline capacity to support *Bifidobacterium* all differ developmentally. Colonization resistance therefore operates in a different context, and a phenotype observed after PEG washout may not reflect the resistance landscape of an AAF-fed infant gut. The experiment demonstrates that a selected, pooled microbial community can confer susceptibility to experimental CDI in this artificial context; it does not establish that AAF feeding caused the microbial differences observed in human participants, nor that those differences cause CDAD in infants.^27^

What experiments would close the gap? We suggest a staged program: (i) individual-donor (non-pooled) FMT with donor-level replication and pre-specified random-effects analysis, to establish whether susceptibility is reproducible across AAF-fed infants; (ii) ecological rescue namely co-transplantation of defined *Bifidobacterium* consortia or HMO supplementation into AAF-BC recipients, to test directly whether restoring the depleted genus restores colonization resistance, i.e., to test the proposed mediation chain; (iii) developmentally congruent recipient models, such as gnotobiotic or antibiotic-conditioned neonatal mice colonized with age-matched infant communities without PEG washout; and (iv) colonization-resistance assays as a more sensitive intermediate readout than disease challenge.^27^ However, none of the above manipulates AAF itself. (v) Direct dietary manipulation: gnotobiotic or humanized juvenile animals colonized with age-matched infant microbiota are randomized to isocaloric, isoproteinic AAF versus NF feeding, followed by CDI challenge and colonization-resistance readouts, ideally in a crossover design to control inter-animal variation. Only this step directly tests whether AAF is the upstream cause of the microbial phenotype, and together with (ii) it would establish the full mediation chain from diet to disease.

### Microbiome alterations and the direction of causality

The authors report compositional differences between CDAD cases and asymptomatic carriers, including depletion of *Bifidobacterium*; beta diversity separated CDAD from carriage, whereas carriers were compositionally closer to healthy controls. As noted above, reduced *Bifidobacterium* with formula feeding is well established and biologically coherent; we do not dispute this finding. The causal question is narrower: does this depletion mediate increased CDAD risk? That specific microbial configurations can influence CDAD susceptibility is plausible and supported by prior work;^28^ ^,^ ^29^ the present cross-sectional data, however, cannot show which configuration is cause and which is consequence.

The clinical microbiome analysis is cross-sectional and cannot establish temporal direction. Reduced *Bifidobacterium* could represent a pre-existing susceptibility phenotype, a consequence of diarrheal disease or intestinal inflammation, or a marker of the underlying condition associated with AAF use. The HMO hypothesis illustrates the general problem: AAF lacks HMOs, and HMOs selectively nourish *Bifidobacterium*, but the original study did not test whether HMO deficiency mediates CDAD risk (e.g., through HMO-supplementation rescue), and the authors themselves acknowledged that AAF differs from human milk in multiple bioactive components beyond HMOs.^30^ Until such mediation is tested, the proposed chain AAF → HMO deficiency → *Bifidobacterium* depletion → CDAD remains mechanistically plausible but unproven. Formally establishing mediation would require measured HMO exposure, the *Bifidobacterium* mediator, and the CDAD outcome within the same longitudinal framework, together with an intervention that perturbs the proposed mediator (e.g., HMO supplementation) or a natural experiment; absent this, the chain remains mechanistically plausible but untested.

## Conclusions

We appreciate the authors' efforts in addressing this clinically important question, and our comments are intended to refine the interpretation of their valuable findings rather than to challenge their observations, a goal aligned with the authors' own appropriately cautious framing. Wang et al. have identified an association between AAF feeding and CDAD that warrants further investigation, but the current evidence does not establish causality, and the dimensions of the analysis (27 events across ~6 predictors) mean the adjusted estimates should be treated as hypothesis-generating.

Clinically, the stakes of this distinction are concrete: in young children, *C. difficile* detection may coexist with asymptomatic colonization, and gastrointestinal symptoms may have multiple causes. Interpreting microbiological findings in isolation, particularly in children receiving AAF for underlying allergic or gastrointestinal conditions, risks creating unnecessary concern and complicating clinical decisions. The present study should stimulate the prospective, adequately powered, carefully adjudicated investigations we have outlined, not discourage or delay AAF use when clinically warranted.

## Data Availability

Data sharing does not apply to this article as no new data were created or analyzed in this study.
